# Decision-Tree-Based Approach for Pressure Ulcer Risk Assessment in Immobilized Patients

**DOI:** 10.3390/ijerph191811161

**Published:** 2022-09-06

**Authors:** Eugenio Vera-Salmerón, Carmen Domínguez-Nogueira, José L. Romero-Béjar, José A. Sáez, Emilio Mota-Romero

**Affiliations:** 1 Servicio Andaluz de Salud, Distrito Sanitario Granada-Metropolitano, Centro de Salud Dr. Salvador Caballero de Granada, 18012 Granada, Spain; 2 Instituto de Investigación Biosanitaria (ibs.GRANADA), 18014 Granada, Spain; 3 Inspección Provincial de Servicios Sanitarios, Delegación Territorial de Granada, Consejería de Salud y Familias de la Junta de Andalucía, 41071 Sevilla, Spain; 4 Department of Statistics and Operations Research, University of Granada, Fuente Nueva s/n, 18071 Granada, Spain; 5 Institute of Mathematics, University of Granada (IMAG), Ventanilla 11, 18001 Granada, Spain; 6 Department of Nursing, University of Granada, Avda. Ilustración 60, 18071 Granada, Spain

**Keywords:** activity, Braden scale, decision trees, immobilized patients, mobility, patient safety, pressure ulcers, skin moisture

## Abstract

Applications where data mining tools are used in the fields of medicine and nursing are becoming more and more frequent. Among them, decision trees have been applied to different health data, such as those associated with pressure ulcers. Pressure ulcers represent a health problem with a significant impact on the morbidity and mortality of immobilized patients and on the quality of life of affected people and their families. Nurses provide comprehensive care to immobilized patients. This fact results in an increased workload that can be a risk factor for the development of serious health problems. Healthcare work with evidence-based practice with an objective criterion for a nursing professional is an essential addition for the application of preventive measures. In this work, two ways for conducting a pressure ulcer risk assessment based on a decision tree approach are provided. The first way is based on the activity and mobility characteristics of the Braden scale, whilst the second way is based on the activity, mobility and skin moisture characteristics. The results provided in this study endow nursing professionals with a foundation in relation to the use of their experience and objective criteria for quick decision making regarding the risk of a patient to develop a pressure ulcer.

## 1. Introduction

Continuous pressure on the skin and/or underlying tissues, or in combination with shearing, causes injury. Such injuries, usually located on bony prominences, are known as pressure ulcers (PUs) [1]. Injuries due to pressure ulcers represent an important health problem, since they have a relevant impact on the mortality and morbidity of immobilized patients. This problem extends to their families, because it involves changes in their quality of life, as well as the affected patients’ lives [2]. Indeed, PUs have a high prevalence in Europe (10.8%) [3]. In Spain, they represent 7% in the hospital environment and 4.79% in the home care setting [4].

Nurses provide comprehensive care to immobilized patients. The increase in life expectancy leads to the appearance of PUs, implying an increase in the number of interventions to be carried out. This results in an increased workload that can be a risk factor for the development of serious health problems, such as burnout syndrome [5].

Quick decision making becomes not only paramount for both the application of preventive measures and adequate distribution of available resources, but also to reduce stress in nursing professionals. Therefore, it is very important to encourage nurses to use their experience to assess the risk of developing PUs in these patients. In this sense, evidence-based nursing healthcare practice or evidence-based nursing (EBN) not only results in the quality and safety of health care for patients [6], but also in their own caregivers. Indeed, there are already studies that, in some way, focus on the risk assessment of developing PUs using one of the subscales of the Braden scale (mainly the activity and mobility subscales [7,8]) in order to reduce the workload associated with the whole scale. Vera-Salmerón et al. [9] justify that the activity and mobility subscales help classification models to discriminate between the risk or no risk of developing PUs that provide safe values of sensitivity and specificity. They also found that the three subscales of activity, mobility and skin moisture provide classification models with sensitivity and specificity values very close to those related to the whole Braden scale even better than the model associated with activity and mobility. The characteristics of activity, mobility and skin moisture can be valued by the nurses only by means of their objective criteria. Therefore, it is very important to provide them with an easy way to assess the risk of PUs only based on their experience but with a solid foundation.

In recent decades, there have been more and more applications where data mining tools have been used in the fields of medicine and nursing [10,11]. These are based on looking for patterns in a dataset to create models that represent its implicit knowledge. Among these techniques, decision trees [12,13] are especially useful in this context, since they are characterized by combining models with high predictive quality and interpretability [14]. Thus, although these models do not in any case replace the diagnosis established by a professional expert, they serve as a support tool in decision making, allowing to ascertain the reasons or key aspects that lead to reaching the output of the model.

Decision trees have been applied with very diverse medical data, such as those related to cancer [15], hepatitis [16] or even COVID-19 [17]. They have also been applied to data associated with pressure ulcers [18,19]. Most of them consider a large set of variables of different types, such as demographics, medications or laboratory tests, from which they create decision trees [20,21,22]. For example, Raju et al. [11] used a multitude of demographic variables, laboratory tests and Braden subscales [23] to analyze risk factors for pressure ulcers. Similarly, Moon and Lee [20] used decision trees on a large dataset considering up to 830 variables on patients in long-term care facilities. Setoguchi et al. [18] applied alternating decision trees to predict pressure injury from variables related to treatment, disease severity, patient mobility and daily activities, concluding that the operation duration, transfer activity and body mass index were the most important.

In this work, two ways for conducting a PU risk assessment based on a decision tree approach are performed. The first way is based on the activity and mobility subscales, whilst the second way is based on the activity, mobility and skin moisture characteristics.

## 2. Patients and Methods

### 2.1. Study Design

A study with an analytical, observational, longitudinal and prospective design was conducted.

### 2.2. Participants

The sample consisted of 16,215 immobilized patients in home and social health care settings older than 64 years with the Braden scale measured and recorded in the Granada-Metropolitan Primary Healthcare District (DSGM) in Andalusia, Spain. The data were collected from the SIRUPP application in the Diraya health history application from the Andalusian Public Health System. The mean age of the participants was 84.13 years (SD = 9.42), and 69.8% of them were female.

### 2.3. Ethical Considerations

The study was carried out in accordance with the 1975 Declaration of Helsinki [24] and was approved by the Clinical Research Ethics Committee at the Andalusian Public Health System (AP-0086-2016).

### 2.4. Methodology

In order to build each classification tree from the data, the *recursive partitioning and regression trees* (RPART) [12,25] algorithm was used. It creates a decision tree by dividing the domain space into different areas that allow for the observations in the dataset to be classified. As a way to perform these divisions, RPART uses a greedy approach that searches all attributes for the value that optimizes a cost function based on reducing the impurity of nodes [12]. The impurity of a node is a measure of the degree of the heterogeneity of the observations it contains, that is, how mixed the class labels are in the node. Thus, a node containing all its observations with the same class has a null impurity.

Following the greedy approach above, RPART selects the best attribute split in terms of impurity reduction, performs the division according to it, and repeats this procedure until a stopping criterion is met. One of the most common criteria for stopping recursive splitting is based on setting the minimum number of observations that a node must contain to be split. Thus, if the number of observations in a node is less than this preset amount, the new split is not carried out, and the node is considered as a final leaf in the tree. It is also common to apply pruning processes to reduce the number of nodes created and increase the generalizability of the model. In this way, the trees are more interpretable, and the risk of overfitting the data is reduced [25]. A widely used pruning procedure is based on the usage of a complexity parameter, which imposes a penalty onto the tree for having too many splits.

The *R* statistical computing software was used for the statistical analysis and the creation of the decision trees. Specifically, each decision tree was created using the *rpart* function (in the *rpart* package) considering at least 20 observations per tree node and a complexity parameter cp=0.01:

> *model* <- *rpart*(*class* ~ ., *data*, *control* = *rpart.control*(*minsplit* = 20, *cp* = 0.01))

with *class* being the output variable, *class* ~ . indicates that *class* was predicted using the rest of the variables, and *data* is the dataset to be used. For each model built, the classification performance estimate, which was calculated using both the classical accuracy and geometric mean, was obtained through 10 runs of a 10-fold cross-validation, averaging the test results. On the other hand, the graphic display of the model was based on the *rpart.plot* function (in the *rpart.plot* package) using the following command:

> *rpart.plot*(*x* = *model*, *type* = 5, *box.palette* = “*RdBu*”, *shadow.col* = “*darkgray*”)

## 3. Results

This section is structured as follows: First, a descriptive analysis of the variables is shown. In Section 3.2, a decision tree for classifying those at risk of PUs was performed based on the activity and mobility subscales. Finally, in Section 3.3, the same approach was used based on the activity, mobility and skin moisture characteristics.

### 3.1. Sample Description

According to the Braden scale scores [23], the individuals were classified into groups at risk or not of developing pressure ulcers. The cut-off point was a score of 18, i.e., a score of 18 or less was considered at risk (mild, moderate, high or severe), whilst a score above 18 was considered safe (not at risk). The descriptive analysis of the subscales is shown in Table 1.

### 3.2. Decision Tree for Pressure Ulcer Risk Assessment Based on Activity and Mobility Subscales

**Figure 1:**
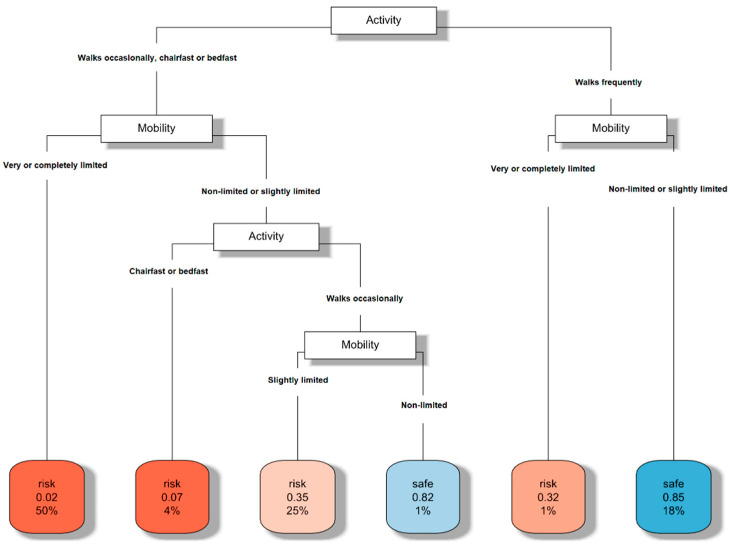
Decision tree for PU risk assessment based on activity and mobility.

The decision tree that classified those at risk of developing PUs based on the activity and mobility subscales is shown in Figure 1. The algorithm used to perform this output highlighted activity as the first characteristic to take into account. Therefore, according to the degree of activity, one had to see the degree of mobility. For instance, if the patient walked frequently (top-right) and their mobility was very or completely limited, they were at risk of developing PUs, whilst if the patient had nonlimited or slightly limited mobility, the tool informed us to no risk of developing PUs. On the other hand, if the patient did not walk frequently and their mobility was nonlimited or slightly limited, the classification depended on the specific degree of activity. Indeed, if the patient was chairfast or bedfast, they were at risk of developing PUs, whilst if the patient walked occasionally and their mobility was slightly limited, they were also at risk. If their mobility was nonlimited, there was no risk. Finally, as expected, if the patient walked occasionally, they were chairfast or bedfast and their mobility was very or completely limited, there existed a risk of developing PUs.

The classification performance estimate was calculated using both the accuracy (*acc* = 0.86) and geometric mean (*gm* = 0.75). There is more relevant information in Figure 1 related to the numbers within each leaf node. For example, the darkest red square (on the far left of the tree) includes two numbers. The first number, 0.02, indicates the percentage of patients in this leaf of the decision tree that were categorized as safe (not at risk), whilst the second number, 50%, provides the percentage of patients in the whole sample with the characteristics of this tree branch. Therefore, as this branch refers to patients that did not walk frequently and with their mobility being very or completely limited, these numbers mean that 50% of the sample had this profile, and 98% of the patients with this profile was categorized at risk. The interpretation of these numbers for each leaf provided realistic information on the validity of the tool. Furthermore, the red color helped with this information, since a darker red color meant a higher percentage of patients classifying at risk in the corresponding leaf of the tree (recall that the first number within the red leaf refers to the percentage of patients classified as safe or not at risk, and, consequently, the complement of this number refers to patients at risk).

### 3.3. Decision Tree for Pressure Ulcer Risk Assessment Based on Activity, Skin Moisture and Mobility Scales

**Figure 2:**
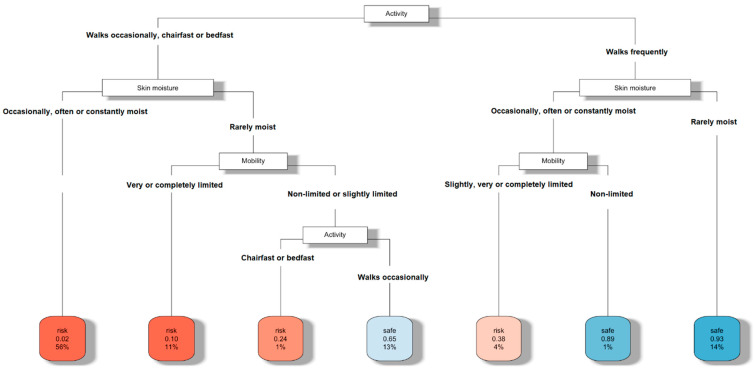
Decision tree for PU risk assessment based on activity, mobility and skin moisture.

The decision tree that classified those at risk or not of developing PUs based on the activity, mobility and skin moisture characteristics of the patients is shown in Figure 2. Similar to the previous scenario, the algorithm used to build the decision tree indicated that activity was the first characteristic that classified those at risk or not. Once the activity was assessed, skin moisture was next and, when necessary, mobility was the last one to take into account. In this situation, characterizing whether or not the patient walked frequently was necessary to assess the moisture characteristic of their skin. Indeed, if the patient walked frequently and their skin was rarely moist, as expected, there was no risk of developing PUs. In addition, if the skin was occasionally, often or constantly moist, but the mobility was nonlimited, there was also no risk of PUs. Furthermore, if the patient rarely had moist skin, with nonlimited or slightly limited mobility and walked occasionally, there was also no risk of PUs. In any other situation, they were classified at risk of developing PUs with different levels of confidence. For instance, if the patient’s profile was in the branch of the decision tree related to if they walked occasionally, were chairfast or bedfast and occasionally, often or constantly had moist skin, then there was a risk of developing PUs without almost any doubt. This was due, as before, to the interpretation of the two numbers within the leaves of the classification tree. In the previous example, 98% of the patients with this profile was classified at risk (the first number, 0.02, always refers to the percentage of patients classified as safe). It was also remarkable, according to the second number, 56%, that this was the percentage of patients with this profile in the whole sample. In order to illustrate a completely opposite situation, if the branch of the decision tree related to patients that walked frequently with rarely moist skin was considered, the first number, 0.93, would indicate that 93% of the patients with this profile was considered safe (not at risk), and they represented, according to the second number, 14% of the whole sample. As before, a darker red color means a higher percentage of patients classifying at risk in the corresponding leaf of the tree.

Finally, the classification performance estimate, calculated using accuracy, was *acc* = 0.90, and the geometric mean was *gm* = 0.88. These values improved those of the previous decision tree.

## 4. Discussion

This work aimed at providing two ways for conducting a PU risk assessment based on a decision tree approach. The first way was based on the activity and mobility subscales, whilst the second one was based on activity, mobility and skin moisture characteristics. With regard to the first, it reflected that activity was the first indicator to consider from objective nursing criteria. It was highlighted that this tool supported the evidence-based nursing healthcare practice since, for instance, as expected, profiles of patients who did not walk frequently with very or completely limited mobility were at risk of developing PUs, and patients who walked frequently with nonlimited mobility were not at risk. Regarding the second way, this tool, as before, highlighted activity as the first patient characteristic to consider, then the skin moisture and, finally, the mobility as the last issue that the nurses had to value. Here, it was also remarkable how this tool corresponded to evidence-based nursing practice. This was supported, for instance, because patients in the profile related to the walking frequently and with rarely moist skin tree branch were considered as safe, i.e., they were not at risk of developing PUs, as expected by a nursing professional with no scale needed. Conversely, a patient within the profile related to not walking frequently who had skin that was occasionally, often or constantly moist was considered at risk of developing PUs.

The use of these decision trees in the area of care, managed by nursing professionals, was intended to serve as a tool for the early detection of the risk of developing pressure ulcers, prompt decision making and the implementation of preventive measures, and, at the same time, to increase patient safety, their quality of life and the efficient management of resources. There is an immediate need to avoid adverse events, such as pressure ulcers. These tools would allow nursing professionals and formal caregivers to implement strategies for the early establishment of preventive measures in some patient clusters, such as institutionalized patients in social health centers or hospitals, where they could be grouped by their conditions of mobility, activity and skin humidity [26]. In addition, it is important that health centers use the mining of their own health data as a useful predictive tool of the appearance of PUs. This would allow for simplified and easy-to-use risk models, specific to their patient population. The early classification of immobilized patients in subgroups that brought together certain characteristics of mobility, activity and/or skin humidity for a quick implementation of preventive measures is an example of this [27]. Different works highlighted pressure ulcer development as one of the main preventable problems and suggested working on it as an avoidable circumstance in a very high percentage of cases. In this sense, providing dynamic and flexible tools for nursing practice that would result in patient safety by preventing adverse events is of great importance [28]. Furthermore, automated risk assessment systems can help save time spent on pressure ulcer prevention and, thus, contribute to better quality care [29].

On the other hand, some works, such as that of Garcia-Sanchez et al. [30], revealed that at-home care with nonprofessional caregivers continues to be the most frequent. In addition, knowledge about risk factors and prevention is insufficient [31]. Thus, when a pressure ulcer appears, these caregivers feel powerless, desperate and suffer. Therefore, it also seems appropriate to provide both patients and their caregivers with health education that provides knowledge beyond their own experiences, and allows them to have a more proactive role in the prevention of these injuries [32]. Simplified and visual tools, such as those presented in this work, could be very useful for the nursing professional in relation to the design of these types of training activities.

The results provided in this study could endow nursing professionals with a foundation in relation to the use of their experience and objective criteria for quick decision making regarding the risk of a patient to develop a pressure ulcer. These nursing criteria, supported by these tools, are very important in order to develop strategies for the prevention of PUs, reducing the health burden associated with this injury and helping to mitigate the workload of these professionals.

### Study Limitations

The limitations of this study were related to the definition of the response variable. The consideration of two levels, risk or not at risk of developing pressure ulcers, allowed for a quick and simplified classification of immobilized patients, but, once the risk was identified, the user did not have enough information on the severity of this risk. It would be advisable to consider this approach with a response variable with more levels. In addition, the consideration of only patients classified as at risk in the first step would be of interest. This approach, with the response variable categorized into two or more levels according to the severity of this risk, would also be advisable for these patients. These are continuing research points for future work.

## 5. Conclusions

This work provided visual and simplified tools for pressure ulcer risk assessment that complement professional knowledge and serve to standardize rapid decision making in patients susceptible to developing this type of injury. This could provide nurses with tools that support, with confidence, their professional criteria based on patient characteristics, such as activity, mobility and skin moisture, which can be interpreted by the trained eye of a professional.

## Figures and Tables

**Table 1:** Description of variables.

*Response Variable*	*Level*	% (*N*)
Pressure ulcer (*N* = 16,215)	(0) No risk	70.0 (11,354)
	(1) Risk	30.0 (4861)
Skin moisture (*N* = 16,215)	(0) Rarely moist	39.3 (6371)
	(1) Occasionally moist	37.9 (6147)
	(2) Often moist	17.0 (2762)
	(3) Constantly wet	5.8 (935)
Activity (*N* = 16,215)	(0) Walks frequently	19.5 (3158)
	(1) Walks occasionally	44.0 (7142)
	(2) Chairfast	26.2 (4249)
	(3) Bedfast	10.3 (1666)
Mobility (*N* = 16,215)	(0) No limitations	8.4 (1357)
	(1) Slightly limited	39.8 (6458)
	(2) Very limited	44.3 (7191)
	(3) Completely immobile	7.5 (1209)

## Data Availability

Data available upon request to the authors.
